# Challenge test studies on *Listeria monocytogenes* in ready‐to‐eat iceberg lettuce

**DOI:** 10.1002/fsn3.1167

**Published:** 2019-09-30

**Authors:** Patrizia Tucci, Gabriella Centorotola, Romolo Salini, Luigi Iannetti, Anna Franca Sperandii, Nicola D'Alterio, Giacomo Migliorati, Francesco Pomilio

**Affiliations:** ^1^ National Reference Laboratory for Listeria monocytogenes Istituto Zooprofilattico Sperimentale dell' Abruzzo e del Molise “G. Caporale” Teramo Italy

**Keywords:** challenge test, *Listeria monocytogenes*, maximum growth rate, minimally processed produce, predictive model

## Abstract

Shelf‐life studies in ready‐to‐eat (RTE) modified atmosphere packaged (MAP) precut iceberg lettuce (minimally processed) were carried out in order to evaluate the natural microflora of the product and survival or multiplication of *Listeria monocytogenes (L. monocytogenes)*, taking into consideration the impact of the production steps resulting in a reduction of the shelf life of the fresh‐cut produce, due to the accelerated enzymatic activity, moisture loss, and microbial proliferation. The research first aimed to evaluate the characteristics of the natural microflora of the product, and then, *L. monocytogenes* dynamics were studied via challenge tests. *L. monocytogenes* concentration was studied at 8 and 12°C storage temperature for 10 days, 6 days longer than their shelf life. The number of *L. monocytogenes* in samples stored both at 8°C and 12°C increased gradually, more evidently in samples stored at 12°C. *L. monocytogenes* dynamics were studied to define maximum growth rate (μmax) at 8°C (0.0104 log_10_CFU/g/h) and 12°C (0.0183 log_10_CFU/g/h). Data obtained from the study were used to develop and validate a specific predictive model able to predict the behavior of *L. monocytogenes* in RTE MAP iceberg lettuce. According to the model, an increase in storage temperature of 6°C (e.g., from 8 to 14°C) would lead to an increase in *L. monocytogenes* concentration of more than 6 log_10_CFU/g at the 10th day of the challenge test (12th days of shelf life). Storage at 4°C allowed to increase *L. monocytogenes* enumeration from 3.30 log_10_CFU/g at D0 to 3.60 log_10_CFU/g at D10. The model could be applied to microorganisms other than *L. monocytogenes*, modifying the coefficients of the polynomial equation on which it is based.

## Graphical abstract

Shelf‐life studies in ready‐to‐eat (RTE) modified atmosphere packaged (MAP) precut iceberg lettuce were carried out in order to evaluate the natural microflora of the product and survival or multiplication of Listeria monocytogenes. Data obtained from the study were used to develop and validate a specific predictive model able to predict the behavior of L. monocytogenes in this product.

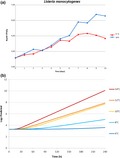

## 1 INTRODUCTION

The demand for convenient foods has increased in the last years, as consumers are interested in buying quality products ready for use. Quite often, minimally processed vegetables are usually used for meals (fresh‐cut fruits, mixed salads etc.). Ready‐to‐eat iceberg lettuce are products that require a certain accuracy in their preparation, trying to guarantee the desired shelf life, and their safety with high sensory and nutritional qualities (Siddiqui & Rahmanand, 2015). Sterility is not guaranteed during the handling of vegetables, and this can reduce the shelf life of the finished product. In fact, due to the moisture loss, accelerated enzymatic activity, and microbial proliferation (Lucera, Costa, Mastromatteo, Conte, & Del Nobile, 2010), cut vegetables are subject to more rapid deterioration processes (Conte, Scrocco, Brescia, & Del Nobile, 2009) and microbial contamination. Despite to the increasing number of foodborne outbreaks caused by the consumption of fresh vegetables, in recent years some studies have focused on observing and predicting the growth of natural microflora and, particularly, pathogens in RTE lettuce (Sant'Ana, Franco, & Schaffner, 2012; Tsironi et al., 2017). Previous reviews have described the survival of pathogenic bacteria such as *E. Coli* O157:H7 and *Salmonella* on different RTE vegetables, but the colonization of lettuce by *Listeria* has received limited attention (Kyere, Palmer, Wargent, Fletcher, & Flint, 2019). *Listeria monocytogenes* is a foodborne pathogen that worries manufacturers of RTE foods because of its wide distribution in the environment and capacity to survive at refrigeration temperatures at low O_2_ levels (Carrasco, Perez‐Rodriguez, Valero, Garcià‐Gimeno, & Zurera, 2008). *L. monocytogenes* has the ability to survive in wet environments for long time without significant decrease in microbial load. A listeriosis outbreak occurring in United States, from July 2015 to January 2016, was linked to a *L. monocytogenes* isolate from packaged salad collected at retail, that was closely related by whole‐genome sequencing (WGS) isolated from ill people (FDA, 2017). Recently, an outbreak of *L. monocytogenes* infections related to frozen corn and probably to other frozen vegetables has been ongoing in five European Union Member States (Austria, Denmark, Finland, Sweden, and the United Kingdom) since 2015. The detection of *L. monocytogenes* indicates that this strain could be persisting in the environment of the processing plant after cleaning and disinfection procedures carried out during periods of no production (EFSA, 2018). In this, just like in other occasions related to different foods (Magalhães et al., 2015; Marini, Magi, Vincenzi, Manso, & Facinelli, 2016; WHO, 2018), the long‐time persistence in the processing environments could be related to the ability to form biofilm on any surface, that leads the bacterium to survive under unfavorable conditions (Meloni et al., 2012). Washing and radiation treatments are able to reduce *L. monocytogenes* colonization; however, these control steps cannot be effective at all times as both bacterial properties and condition of lettuce plants are important factors to consider (Kyere et al., 2019). The European Union Food Law identifies the Food Business Operator (FBO) as the principal responsible for his production process, and accordingly, he required to evaluate, by means of documented scientific methods, the hazards to human health related to the consumption of his own products. In addition, the FBO must know the risks associated with its production, has to evaluate the foods and determine its durability under foreseeable and correct storage conditions along the entire food chain (shelf life), and included domestic storage time. To set up the shelf life of their products, FBOs could perform microbiological challenge tests, to validate the entire production process and storage of the foodstuffs. Furthermore, the possibility of carrying out challenge tests is expressly provided by EC Regulation 2073/2005, which requires the FBO to ensure compliance with the microbiological criterion for *L. monocytogenes* of 100 cfu/g in foods that support the growth of this microorganism also by carrying out studies "to evaluate the development or survival of the microorganisms in question that may be present in the product during production and the shelf life, under reasonably foreseeable conditions of distribution, storage" (Regulation (EC) No. 2073/2005). A microbiological challenge test assessing maximum growth rate (μmax) of *L. monocytogenes* in a food is a laboratory‐based study able to calculate the rate of the growth in an artificially contaminated food stored at a defined temperature. The growth of *L. monocytogenes* in foods is linked to many factors such as stress applied to the strains, the characteristics of the strains, NaCl content, pH, water activity (a_w_), food composition, in association with microflora, antimicrobial constituents, and extrinsic properties like packaging gas atmosphere and temperature storage (EURL, 2014). The present study was a part of a research project founded by the Italian Ministry of Health, to assess the risk for the Italian consumer to various pathogens contaminating ready‐to‐eat products (RTE) not submitted during processing to steps able to ensure microbiological safety, as RTE fresh vegetables packaged in modified atmosphere (MAP). The aim of this work was to carry out a challenge test in a RTE iceberg lettuce to study the kinetics of natural microflora and *L. monocytogenes,* that frequently contaminates vegetables and whose survival is strongly influenced by the product's storage temperature. Based on the data obtained from challenge tests, a mathematical model was developed and applied, useful to predict *L. monocytogenes* growth kinetics in RTE fresh vegetables during their shelf life, assuming different temperatures of storage as reported by Iannetti et al. (2017) for food of animal origin.

## 2 MATERIALS AND METHODS

### 2.1 Experimental plan

For this study, a total of 94 minimally processed packed iceberg lettuce (weighing 100 g each) were bought at retail on day one of production. All the packages belonged from the same production batch. Samples were transported at 4 ± 2°C to the laboratory located at the Istituto Zooprofilattico Sperimentale dell'Abruzzo e del Molise (IZSAM) and stored at 4 ± 2°C for 24 hr. The challenge test lasted 10 days, storing the iceberg lettuce at 8°C and 12°C, 6 days longer than their shelf life. To carry out the study, 68 samples were contaminated with *L. monocytogenes*, and 34 were stored at 8 ± 2°C and 34 at 12 ± 2°C; conversely, the last 26 packs were used as control samples, and 13 were stored at temperature of 8 ± 2°C and 13 at 12 ± 2°C.

### 2.2 Preparation of the inoculum

A suspension of two different strains was used as inoculum, composed by the reference strain ATCC 7644 and the EURL strain 12M0B098LM (Guillier, Lardeux, Michelon, & Ng, 2013). The preparation of the inoculum was done following the European Reference Laboratory for *L. monocytogenes* guideline (EURL, 2014) with some modifications (concentration of the inoculum and use of a mix of two *L. monocytogenes* strains). The challenge test was initially carried out to assess the growth potential, and then, it seemed more interesting to calculate the maximum growth rate, knowing that the use of two *L. monocytogenes* strains, instead of one, would not have influenced the result of the study. The strains, stored in Microbank™ (Pro‐Lab Diagnostics) at −80°C, were individually revitalized at 37 ± 1°C for 18h in BHI broth (Brain Heart Infusion, Sigma‐Aldrich Co. LLC.). In order to get adapted to the storage temperature of the product, new subcultures were set up in BHI broth and incubated at 8 ± 1°C and 12 ± 1°C until reaching the stationary phase of each bacterial strain (EURL, 2014; NACMCF, 2010). The inoculum suspension was then prepared, consisting of equal parts of liquid cultures of the two selected strains. The mixture was enumerated according to ISO 11290‐2:1988/Amd. 1:2004. The suspension was subsequently diluted (saline water) to obtain a concentration of 10^3^ CFU/g in the product.

### 2.3 Contamination method

**Figure 1:**
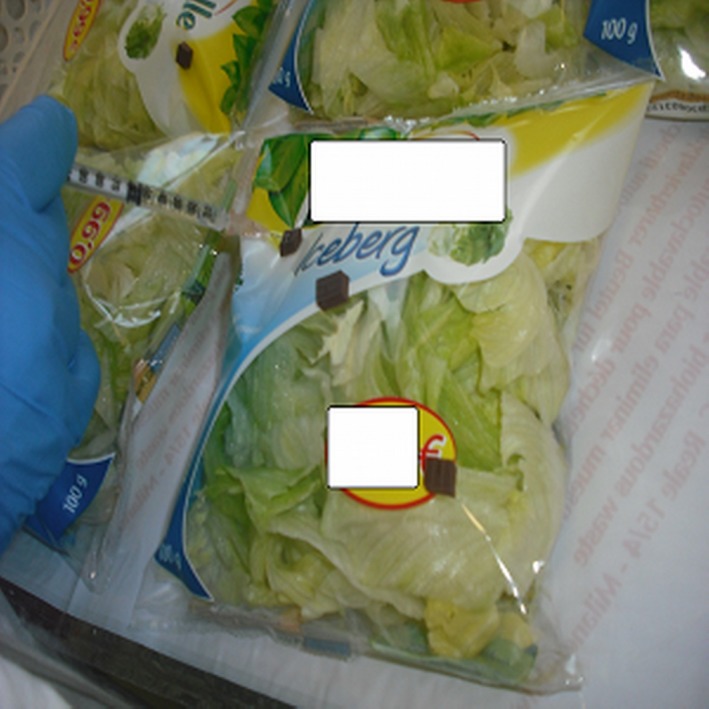
Contamination with septum method in 3 points

Contamination of the packages was done accordingly with the Guideline of the European Reference Laboratory for *L. monocytogenes* (EURL, 2014). The contamination was performed with the septum method; three points (Figure 1) of inoculation were identified, and on each point, a rubber septum was placed. The contamination of the packages was carried out with a syringe, distributing 1 ml of mixed bacterial suspension (0.3, 0.3, and 0.4 ml to a 1% of the weight of the products). Each septum was then sealed with another septum to assure the closure of the hole of the package. To ensure homogeneous contamination within the product, the packs were shaken manually for 1 min. Equal amount of saline water (IZSAM) was injected in control samples.

### 2.4 Samples preparation and analytical determinations

The samples were tested at preset times (D = days), up to the twelfth day after production. D0 was the day of contamination, carried out two days after production (the shelf life of the product was 8 days and 4 days of extended shelf life). At each sampling time, 2 packs were analyzed for each storage temperature. In contaminated samples, sampling was performed once at D0, twice a day (at 8 a.m. and at 5 p.m.) from D1 to D6, and once from D7 to D10 and tested for detection and enumeration of *L. monocytogenes* according to ISO 11290‐1:^96^/Amendment 1:2004 and ISO 11290‐2:^98^/Amendment 1:2004, respectively. The enumeration of *L. monocytogenes* in the contaminated samples was done by adding 100 ml of diluent (Peptone Water, Oxoid) to 100 g of product, and after homogenization with stomacher (Stomacher 400 Circulator, International pbi) for 2 min, three laboratory samples of 25 g of each diluted quantity were added to peptone water (final weight/volume of 1/10). Decimal dilutions of the initial suspension were spread onto the media. The use of three subsamples was applied to obtain a better repeatability of the results. Control samples were tested at D0, D2, D3, D5, D7, D9, and D10 for detection and enumeration of *L. monocytogenes* and for the enumeration of total viable bacteria at 30°C (ISO 4833:^2003^), *Enterobacteriaceae* (ISO 21528‐2:^2004^), lactic acid bacteria (ISO 15214:^1998^), yeast and molds (ISO 21527‐1:^2008^), coagulase‐positive staphylococci (ISO 6888‐2:^1999^/Amendment 1:2003), *Pseudomonas* spp. (AFNOR NF V 04–504:^2006^), and *Bacillus cereus* (ISO 7932:^2004^). The enumeration of *Micrococcaceae* was also carried out on control samples, following an internal method. The decimal dilutions were spread on Baird‐Parker agar (Biolife) and incubated at 37 ± 1°C for 48 ± 2 hr. After incubation, the typical colonies (black, glossy black, or gray) were counted, and five colonies were then isolated on plates of blood agar and subjected to confirmatory tests (Gram staining and glucose fermentation). The pH was measured according to ISO 1842:^91^, and a_w_ determination was performed with AquaLab 4 TE (METER Group, Inc.), in accordance with ISO 21807:^2004^. For a_w_ determinations, 50 g of sample was cut and homogeneously transferred into a plastic cup. Then, the cups were inserted into the measuring probe.

### 2.5 Calculation of maximum growth rate (μmax) and Predictive model

The calculation of maximum growth rate (μmax in natural logarithm) at 8 and 12°C was done using DMFit software (ComBase, 2018a). Prediction of *L. monocytogenes* concentrations at 4, 8, 12, 14, and 16°C was done according to Iannetti et al. (2017), but considering a stable, growth supporting environment for the whole duration of the retention period. Fresh vegetables are characterized by a very high water activity during the entire storage period and a pH that does not undergo large variations. Therefore, in this case the environmental conditions were ideal for the development of *L. monocytogenes* during the entire period of conservation, which was relatively limited and therefore not sufficient to induce a depletion of food resources and thus to generate competition with the autochthonous microbial flora. It was therefore sufficient to consider a only growth secondary predictive model, based on the application of the simple polynomial model (1) for the estimation of the change in the growth rate μ with changes in environmental conditions, in this case the only storage temperature, the pH, and the bw being static.

The polynomial model applied was as follows:

(1) Ln=a0+a1·T+a2·pH+a3·bw+a4·T·pH+a5·T·bw+a6·pH·bw+a7T2+a8·pH2+a9·bw2

In particular, for the prediction of *L. monocytogenes* growth, the primary model from Baranyi and Roberts (1994) was used, taking into account the value h0, indicative of the previous cell history, for the definition of the duration of the lag phase. The effects of the environment were added through a simple polynomial secondary model where the environment is characterized by three variables: temperature (T), pH, and bw $b_{w}=\sqrt{1-a_{w}}$. The last variable is a modified version of water activity (a_w_) according to Gibson, Baranyi, Pitt, Eyles, and Roberts (1994).

The coefficients of the model (a0, a1, a2...) were specific for *L. monocytogenes*, drawn from ComBase (Combase, ), and were derived from experiments carried out in culture broths where the microorganisms found the best growth conditions. The effect of the difference between the growth rate obtained from the model (1) based on coefficients determined by the culture broth and the actual growth rate found in RTE salads was considered by applying a bias factor (Baranyi, Pin, & Ross, 1999). In the model was developed a lettuce/broth culture bias factor greater than the most probable real condition, necessary to consider a "worst‐case scenario" situation (bias factor used = 0.4). The value h0, indicative of the speed of adaptation of the microbial cells to the new environment, was fixed at 4 for *L. monocytogenes.* The programming of an Excel spreadsheet (Excel 2010, Microsoft Corporation, USA) allowed to obtain the observed kinetics of *L. monocytogenes* at 8°C and 12°C and the predicted at three different temperatures of storage (4°C, 14°C and 16°C). The presence of statistical differences between observed and predicted values at 8°C and 12°C was evaluated with a Wilcoxon matched‐pairs signed‐rank test.

## 3 RESULTS AND DISCUSSION

The experiment allowed to evaluate the trend of chemical–physical characteristics and microbiological dynamics in RTE, MAP iceberg salad at two different temperatures (8°C and 12°C).

### 3.1 Chemical–physical analysis

The determination of a_w_ has given almost constant results until the end of the challenge test. In the packs stored at 8°C, the mean value of a_w_ at D0 was 0.998 ± 0.001. In all samples, the trend was constant up to D5, but at D7 a gradual decrease was detected at both temperatures. At the end of the challenge test (D10), the mean value of a_w_ obtained was 0.995 in samples stored at 12°C and 0.998 in those stored at 8°C. The pH was in average at values around 6.5, with a swinging trend: The value was at D0 6.85 ± 0.05, and then, the values showed at D2 a decrease at 8°C (6.37 ± 0.01) and at 12°C (6.48 ± 0.01), and later, the values increased to 6.81 ± 0.02 in samples stored at 8°C and to 6.63 at D10 in samples stored at 12°C.

### 3.2 Microbiological analysis

**Figure 2:**
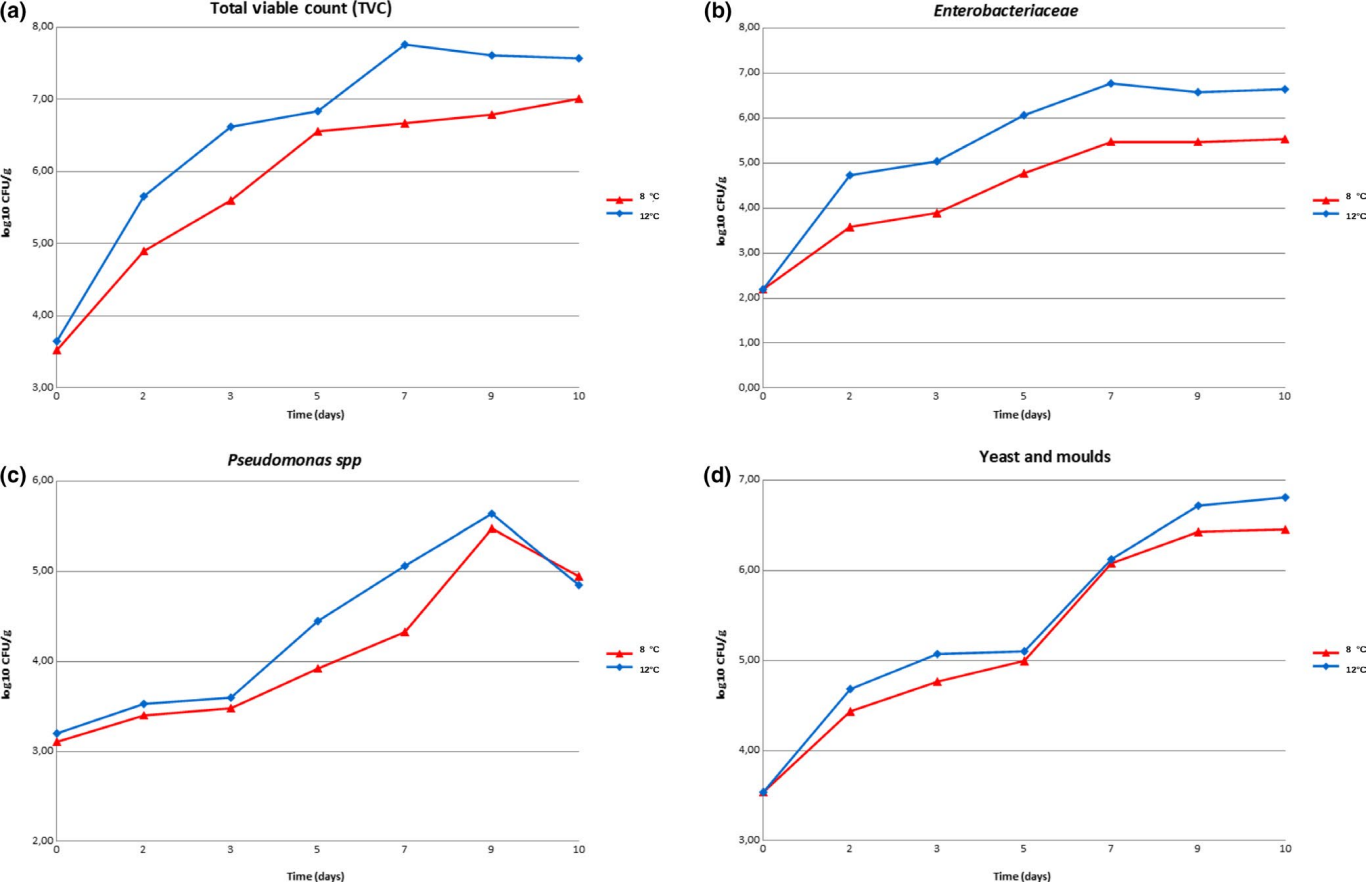
(a) Results of TVC in the control batch of iceberg lettuce stored at 8 and 12°C. **2** (b) Results of *Enterobacteriaceae* concentration in the control batch of iceberg lettuce stored at 8 and 12°C. **2** (c) Results of *Pseudomonas* spp concentration in the control batch of iceberg lettuce stored at 8 and 12°C. **2** (d) Results of yeast and molds concentration in the control batch of iceberg lettuce stored at 8 and 12°C

**Figure 3:**
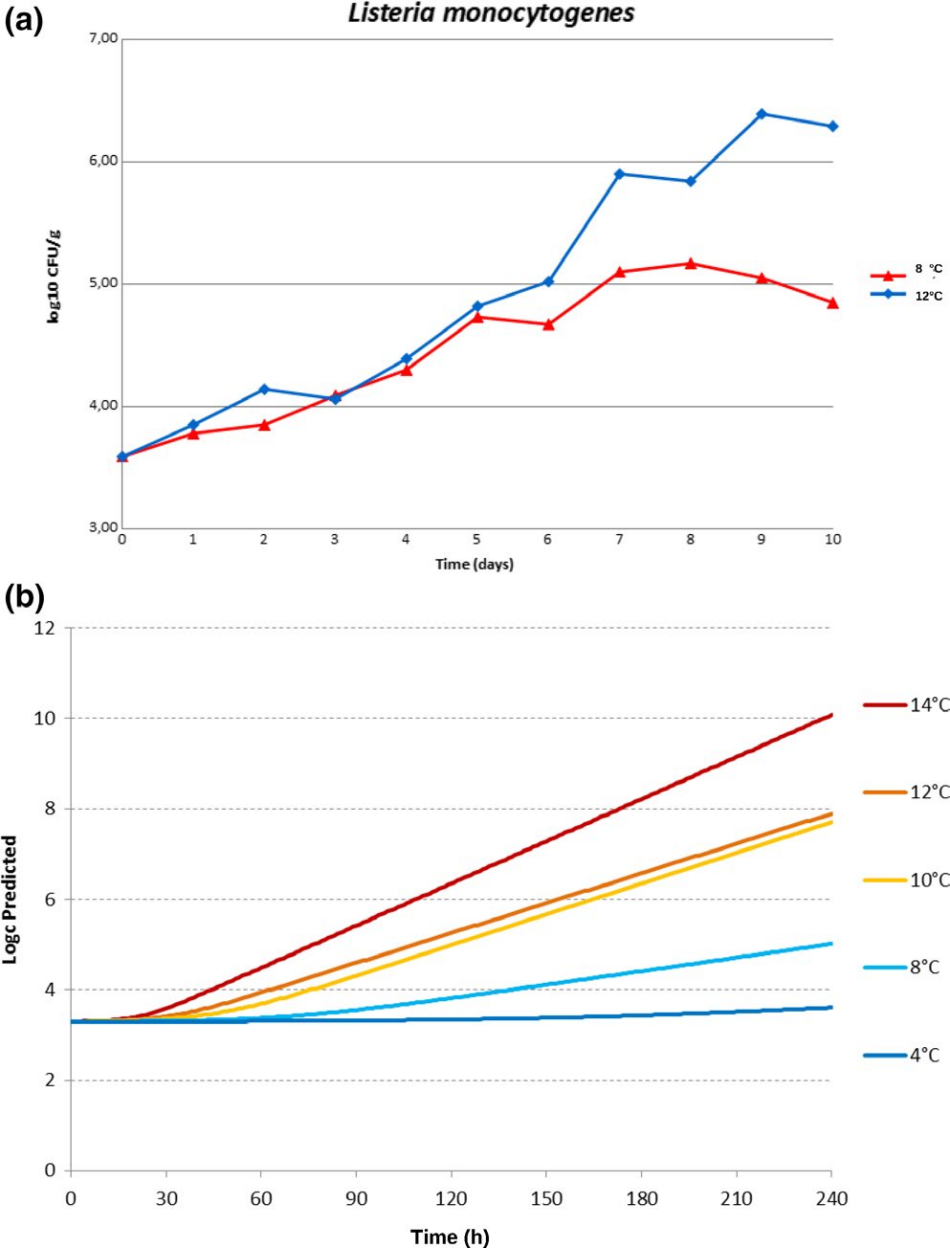
(a): Results of *Listeria monocytogenes *concentration in the contaminated batch of iceberg lettuce stored at 8 and 12°C. **3** (b) Predicted growth predictions of *L. monocytogenes* (log_10_CFU/g log) at 4, 8, 10, 12 and 14°C in the iceberg lettuce

The *Listeria monocytogenes *counts obtained from the inocula were 4.51 log_10_CFU/ml for the samples stored at 8°C and 4.63 log_10_CFU/ml for the samples stored at 12°C. Contamination levels higher than those indicated in literature for naturally contaminated RTE vegetables were used. Little et al. (2007), in their study, found a maximum level of contamination of 990 CFU/g of *L. monocytogenes* in a sample of prepackaged mixed raw vegetable salad. Soderqvist (2017) in a study on the bacterial pathogens in RTE prepacked mixed‐ingredient salads reported that in the UK and in Ireland, only 0.1%–0.3% of tested samples were contaminated by *L. monocytogenes* with levels ranging from 170 to 1,200 CFU/g. Regarding microbiological parameters, during storage, microbial proliferation was observed, especially at 12°C, similar to those found in other studies on RTE vegetables (Carrasco et al., 2008). In all tested samples, *Micrococcaceae*, *Bacillus cereus,* and positive coagulase staphylococci concentrations were below the detection limit of the method (10 CFU/g). Also, lactic acid bacteria (LAB) concentrations were lower than the detection limit, in contrast with Carrasco (2008) that found an initial concentration of LAB between 1.6 and 3 log_10_CFU/g in RTE iceberg lettuce. This difference may be due to the fact that the modified atmosphere inside the packages could select bacteria able to grow under such atmosphere. The trends of total viable count (TVC) are reported in Figure 2. The mean values of the TVC obtained at D0 were 3.52 ± 0.08 log_10_CFU/g. Other authors reported TVC between 10^4^ and 10^6^ CFU/g (King, 1991). In samples stored at 8°C, TVC increased slowly from D2 along the entire storage period reaching at D10 a mean concentration of 7.01 ± 0.03 log_10_CFU/g. In the samples stored at 12°C, a higher TVC was observed, reaching an average value of 7.57 ± 0.13 log_10_CFU/g at D10 (Figure 2a). The *Enterobacteriaceae* count was 2.19 ± 0.15 log_10_CFU/g at the beginning of the trial, similar to the level observed by Skalina (2010) that was from 1.7 to 1.9 log_10_CFU/g. The trend of *Enterobacteriaceae* in samples stored at 8°C had a slight increase throughout the experiment, reaching an average value of 5.53 ± 0.03 log_10_CFU/g at D10. At 12°C, a faster increase in *Enterobacteriaceae* concentration was observed, reaching at D10 average values of 6.64 ± 0.06 log_10_CFU/g (Figure 2b). Similarly, Rocourt (2003) observed a high increase in the level of *Enterobacteriaceae* at D7, especially in samples stored at 12°C. Different species of *Enterobacteriaceae* such as *Pantoea* spp., *Serratia* spp., *Enterobacter* spp., and *Klebsiella* spp. were isolated from the samples. At D0, the mean values of *Pseudomonas* spp. concentrations found were 3.20 ± 0.17 log_10_CFU/g. The trends of *Pseudomonas* spp. are reported in Figure 2c. Evaluation of the results obtained at 8°C and 12°C for the enumeration of *Pseudomonas* spp. highlighted an increase in concentration. These microorganisms, because of their psychrophysiology, can pose a risk for the consumer. The trend of *Pseudomonas* spp. increased during the shelf life test reaching at D10 mean values of 5.41 ± 0.06 log_10_CFU/g in salads stored at 8°C and 5.63 ± 0.05 log_10_CFU/g in salads stored at 12°C. The trends of yeasts and molds are reported in Figure 2d. The average concentration of yeasts and molds at D0 was 3.53 ± 0.21 log_10_CFU/g. During the storage period, for the two temperatures considered, a continuous and gradual increase in the concentration of yeasts and molds was observed until D10. At D10, the mean value found was 6.45 ± 0.07 log_10_CFU/g in samples stored at 8°C and 6.81 log_10_CFU/g ± 0.04 log_10_CFU/g in samples stored at 12°C. *L. monocytogenes* was not found in control samples. In Figure 3a, concentration of *L. monocytogenes* in artificially contaminated samples is reported. At D0, the mean values found were 3.59 ± 0.06 log_10_CFU/g. At D7 and at D8, there was an increase in about 1.5 log_10_CFU/g (5.10 ± 0.06 log_10_CFU/g), while at 12°C it was of about 2.3 log_10_CFU/g (5.90 ± 0.05 log_10_CFU/g). The trend of *L. monocytogenes* in samples showed a gradual increase, to a maximum of 5.20 log_10_CFU/g at D8 in samples stored at 8°C and to 6.43 log_10_CFU/g at D9 in those stored at 12°C. Noor (2015) conducted a challenge test study on chili, onion, capsicum, and coriander, contaminating these with various bacteria including *Listeria* spp. and preserving these at room temperature. After 15 days of observation, the microbial growth was declined from the initial load so the survival of pathogenes in vegetable samples raises the necessity of maintaining proper sanity condition during handing and storaging. In chili samples, the bacterial load was reduced more than 2 log, in onion samples nearly 3 log, in capsicum samples more than 2 log, and in coriander samples nearly 3 log. Carrasco (2008) observed an increase of 3 log CFU/g of *L. monocytogenes* in RTE MAP lettuce stored at 13°C after 7 days of storage, and of 4.85 log_10_CFU/g after 14 days, with no lag time. A slight decrease in the concentration of *L. monocytogenes* was observed at the end of the study, reaching at D10 an average value of 4.85 ± 0.08 log_10_CFU/g at 8°C and 6.29 ± 0.03 log_10_CFU/g at 12°C. The maximum growth rate (μmax) calculated at 8°C was 0.0104 ± 0.00184 log_10_CFU/g/h and at 12°C was 0.0183 ± 0.00223 log_10_CFU/g/h.

### 3.3 Predictive model

As determined by the shelf‐life studies, the iceberg lettuce was characterized by a very high, stable a_w_, considered for simplicity of 1, throughout the experiment. Even pH showed no significant variation, with an average value of 6.5–6.6. Therefore, environmental conditions were suitable for the development of *L. monocytogenes* during the entire retention period, which was relatively limited and not sufficient to induce a depletion of trophic resources and thus to compete with native microbial flora. It was first considered a primary model for the definition of the maximum growth rate (μmax) and the lag phase, and later, it was estimated by a secondary model how the growth rate was influenced by the temperature of preservation, being static the pH and a_w_. The difference between the values of the mathematical model and those observed at 8°C and 12°C was minimal (<0.5 log_10_ UFC/g). The Wilcoxon matched‐pairs signed‐rank test confirmed the absence of statistically significant differences (*p *> .05) between observed and predicted values both at 8°C (*p* = .85716) and at 12°C (*p* = .9124). The model was therefore considered applicable in a satisfactory way to predict growth kinetics in RTE MAP lettuce during shelf life up to D10, at different temperatures. Figure 3b shows the predictions for the concentration of *L. monocytogenes* in RTE salads at 4°C, 10°C, and 14°C. According to this model, an increase in storage temperature of 6°C (from 8 to 14°C) would lead to concentration of *L. monocytogenes* at D6 (144 hr) almost 3 log_10_CFU/g higher compared with 8°C. Conversely, storage at 4°C would allow to increase the number of *L. monocytogenes* from 3.30 log_10_CFU/g at D0 to 3.60 log_10_CFU/g at D10, with a growth of only 0.3 log_10_CFU/g.

## 4 CONCLUSIONS

Raw vegetables are inevitably contaminated by spoiling and pathogenic microorganisms during primary stage (cultivation) caused by poor hygienic handling, processing and storage practices. The potential of microbial populations to proliferate and cause bad organoleptic quality and safety issues is mostly linked to the temperature throughout the entire production and supply chain. Based on the current evolution of the dietary habits of consumers, raw vegetable produce, such as RTE salads, are strongly recommended by the current guidelines for a healthy and balanced diet with high daily intake (USDHHS/USDA, 2015). The data obtained show the utmost importance of the hygiene of the product, as indicated on the label, without further washing. From this point of view, the study contributes to the definition of the characteristics of the microflora, to better define the hygienic working practices, and to evaluate the trend of *L. monocytogenes* (maximum growth rate) during storage at 8°C and 12°C. During storage, the concentration of *Enterobacteriaceae*, yeast, molds, and *Pseudomonas* spp. increased, especially for RTE stored at 12°C. These microorganisms, especially *Pseudomonas* spp. in such products, can pose a risk to the consumer health. Based on the data obtained, RTE iceberg lettuce supports the growth of *L. monocytogenes,* and therefore, for consumer safety, it is advisable to store the product at a temperature of 4°C to limit bacterial proliferation. As confirmed by predictive models, in the case of abuse temperature as generally occurs in domestic kitchen, this product should be stored at maximum at 8°C (including storage at retail), for a period not exceeding 6 days from the date of production. Therefore, any prolonged thermal abuse in the distribution chain or even domestically could give rise to a significant increase in the risk for the consumer. This is true also in case of RTE produce with less than 5 days of shelf life, which are currently not considered as risk products for *L. monocytogenes* according to EU legislation. Performing challenge tests on RTE products should be highly recommended, also in order to generate data for the production and validation of predictive models, as valuable tool to be used in risk analysis activities.

## 5 CULTURE MEDIA

Aloa (Agar Listeria according to Ottaviani and Agosti) (BD Difco Oxford, United Kingdom).

Fraser broth + listeria fraser supplement (Biolife Italiana srl, Milan, Italy + Liofilchem, Roseto degli Abruzzi, Teramo, Italy).

Fraser‐demi broth + listeria fraser supplement (Biolife Italiana srl, Milan, Italy + Liofilchem, Roseto degli Abruzzi, Teramo, Italy).

Oxford Agar (BD Difco Oxford, United Kingdom).

Plate Count Agar Standard (Panreac, Cinisello Balsamo, Milan, Italy).

Violet Red Bile Glucose Agar (VRGB) (Oxoid Ltd, United Kingdom).

Nutritive Agar (Oxoid Ltd, United Kingdom).

MRS/De Man, Rugosa e Sharpe a pH 5.7 ± 0.1 (Biolife Italiana srl, Milan, Italy).

Dichloran‐Rose Bengal Chloramfenicol (DRBG) (Oxoid Ltd, United Kingdom).

Baird‐Parker Agar (Biolife Italiana srl, Milano, Italy).

BHI Broth (Brain‐heart infusion broth—Sigma‐Aldrich Co. LLC., St. Louis, Missouri, USA).

Cetrimide‐fucidin‐cefaloridine agar (Oxoid Ltd, United Kingdom).

Kligler‐Hajna Agar (Biolife Italiana srl, Milan, Italy).

Buffered peptone water at pH 7.0 ± 0.2 (Biolife Italiana srl, Milan, Italy).

Blood Agar (Biomerieux, Marcy‐l'Etoile, France).

Culture media for sugars: ramnosio (Biolife Italiana srl, Milan, Italy).

Culture media for sugars: xilosio (Sigma‐Aldrich Co. LLC., St. Louis, Missouri, USA).

## CONFLICT OF INTEREST

The authors declare that they do not have any conflict of interest.

## ETHICAL APPROVAL

This study does not involve any human or animal testing.

## INFORMED CONSENT

Written informed consent was obtained from all participants.
